# Development and Validation of Chronopotentiometric Method for Imidacloprid Determination in Pesticide Formulations and River Water Samples

**DOI:** 10.1155/2016/5138491

**Published:** 2016-03-06

**Authors:** Ana Đurović, Zorica Stojanović, Snežana Kravić, Nada Grahovac, Vojislava Bursić, Gorica Vuković, Zvonimir Suturović

**Affiliations:** ^1^Faculty of Technology, Department of Applied and Engineering Chemistry, University of Novi Sad, Bulevar Cara Lazara 1, 21000 Novi Sad, Serbia; ^2^Institute of Field and Vegetable Crops, Maksima Gorkog 30, 21000 Novi Sad, Serbia; ^3^Faculty of Agriculture, University of Novi Sad, Trg Dositeja Obradovića 8, 21000 Novi Sad, Serbia; ^4^Institute of Public Health, Bulevar Despota Stefana 54a, 11000 Belgrade, Serbia

## Abstract

A new electrochemical method for determination of imidacloprid using chronopotentiometry on thin film mercury and glassy carbon electrode was presented. The most important experimental parameters of chronopotentiometry were examined and optimized with respect to imidacloprid analytical signal. Imidacloprid provided well-defined reduction peak in Britton-Robinson buffer on thin film mercury electrode at −1.0 V (versus Ag/AgCl (KCl, 3.5 mol/L)) and on glassy carbon electrode at −1.2 V (versus Ag/AgCl (KCl, 3.5 mol/L)). The reduction time was linearly proportional to concentrations from 0.8 to 30.0 mg/L on thin film mercury electrode and from 7.0 to 70.0 mg/L on glassy carbon electrode. The detection limits were 0.17 mg/L and 0.93 mg/L for thin film mercury and glassy carbon electrode, respectively. The estimation of method precision as a function of repeatability and reproducibility showed relative standard deviations values lower than 3.73%. Recovery values from 97.3 to 98.1% confirmed the accuracy of the proposed method, while the constancy of the transition time with deliberated small changes in the experimental parameters indicated a very good robustness. A minor influence of possible interfering compounds proved good selectivity of the method. Developed method was applied for imidacloprid determination in commercial pesticide formulations and river water samples.

## 1. Introduction

In the past several decades, due to the progress in worldwide agricultural production, use of pesticides has been significantly increased. Increased public concern about the dietary risks of pesticides led to a major change in pesticide law. As a result, current pesticide policy in Europe and America is focused on reducing pesticide applications [1, 2]. Nevertheless, some of these chemicals are very persistent, which led to their growing presence in the environment (crops, soil, and water). Thereby, analytical methods concerned with pesticide levels in environmental samples have attracted significant attention.

**Figure 1:**
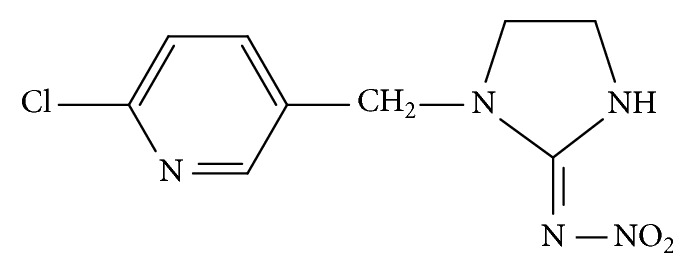
Structural formula of imidacloprid.

Imidacloprid [1-6(Chloro-3-pyridylmethyl)-N-nitroimidazolidin-2-ylideneamine] (Figure 1) is the most commonly used member of neonicotinoid group of insecticides developed in the early 1990s. Since its discovery and utilization, it became the most widely used insecticide for pest control on a broad range of crops [3]. As agonist of the postsynaptic nicotinic acetylcholine receptors, imidacloprid selectively acts on the insect's central nervous system, with much lower toxicity to mammals [4, 5]. Still, numerous concerns exist about its use. Recent studies indicated that widespread agricultural use of imidacloprid and other neonicotinoids may be contributing to decline of honey bee and bumble bee colonies [6–9]. In addition, extreme sensitivity of many aquatic species [10–15] and birds [16] towards imidacloprid was noticed. Moreover, its chemical properties such as high water solubility and long half-life in soil and water, in the absence of light, contribute to its environmental persistence and susceptibility to transport into aquatic ecosystems through runoff and drainage of agricultural areas [10, 17]. Thus, increased concentrations of this insecticide in environmental water samples were detected in many countries worldwide [18–24] with highest reported concentration of 0.32 mg/L in Netherlands [25]. However, lacking systematic environmental monitoring in most countries, due to expensive and time-consuming analytical methods for pesticide analysis, these data are incomplete. Consequently, development of rapid and low cost methods for pesticide analysis of environmental water samples is of crucial importance.

Techniques based on liquid chromatography have been mostly applied for imidacloprid determination in water samples [15, 20, 26–29]. Nowadays, liquid chromatography with mass spectrometry detection represents the most selective and sensitive technique, allowing the identification of pesticide residues at very low levels. However, the high price of HPLC–MS instrumentations still presents an obstacle for its wide use, especially in developing countries. Recently reported data showed that liquid chromatography with UV detection [30] and ELISA [31] could be used as cheaper and still valid analytical procedures for pesticide determination. Even though these techniques are routine in pesticide determination, bulky nonportable instrumentation, long analysis time, and complicated sample preparation make them unsuitable for in-field analysis.

In the past few decades, electrochemical methods have received increasing attention for pesticide determination, due to their speed, simplicity, sensitivity, and more feasible analysis [32]. Numerous studies about electrochemical determination of imidacloprid based on its reduction have been reported. Imidacloprid reduces in two steps, by capturing four electrons in the first step and two in the second and producing the hydroxylamine and amine derivatives, respectively [33]. Differential pulse polarography [33] and square wave adsorptive stripping voltammetry [34] using hanging mercury drop electrode (HMDE) were among first electrochemical approaches applied for imidacloprid determination in commercial formulations and river water samples. Recently, the use of voltammetry on glassy carbon electrode (GCE) [35], carbon paste electrode (CPE) [36, 37], bismuth film electrode (BiFE) [38], and silver-amalgam film electrode (Hg(Ag)FE) [39] was described. In order to increase sensitivity and selectivity in the imidacloprid determination, different modification procedures of usual electrodes were applied. These modified electrodes include Prussian blue multiwalled carbon nanotubes modified GCE (Prussian blue/MWNT/GCE) [40], poly(carbazole)/chemically reduced graphene oxide modified GCE (PCz/CRGO/GCE) [32], nanosilver Nafion®/nano-TiO_2_ Nafion modified GCE (nAgn_f_/nTiO_2_n_f_/GCE) [41], *β*-cyclodextrin polymer functionalized reduced-graphene oxide modified GCE (*β*-CDP/rGO/GCE) [42], imprinted poly(o-phenylenediamine) membranes at reduced graphene oxide modified electrode (Imprinted PoPD-RGO/GCE) [43], and copper(II) phthalocyanine modified carbon ceramic electrode (CuPc/GCE) [44]. Application of modified electrodes, unlike bare GCEs, although having a lower detection limit, mainly involves long and complicated preparation procedures, leading to greatly prolonging the analyses.

A detailed literature review [32–44] indicated that application of chronopotentiometry in imidacloprid determination has not been reported until now. Thereby, this study is concerned with utility of chronopotentiometry with use of thin film mercury electrode (TFME) as a working electrode for quantification of imidacloprid content in pesticide formulations and river water samples. The proposed method was validated in terms of linearity, LOD, LOQ, precision (repeatability and repeatability), selectivity, accuracy, and robustness according to US EPA guidelines [45]. Additionally, chronopotentiometry with GCE was applied for imidacloprid determination as well and was used for comparison purposes. Unlike modified GCEs that have been mostly implied for imidacloprid determination, electrodes applied in this study do not require complicated preparation procedures.

## 2. Experimental

### 2.1. Chemicals and Reagents

Imidacloprid analytical standard (>99.1%) was purchased from Dr. Ehrenstorfer, Augsburg, Germany. Standard stock solution of imidacloprid (0.40 g/L) was prepared by dissolution of solid standard in distilled water. The solution was stable for a period of three weeks if stored in the dark at 4°C. Orthophosphoric, boric, and acetic acids of analytical grade were provided by Lach-Ner (Brno, Czech Republic). HPLC grade methanol and analytical grade hydrochloric acid, sulphuric acid, and sodium sulphite were purchased from Merck (Darmstadt, Germany). Sodium hydroxide was of analytical grade (Donau Chemie, Wien, Austria). Studied supporting electrolytes were citrate buffer, phosphate buffer, Britton-Robinson (BR) buffer, acetate buffer, hydrochloric acid, sulphuric acid, and sodium sulphite solutions. BR buffer was prepared from equimolar 0.04 mol/L stock solutions of orthophosphoric, boric, and acetic acids. Required pH value of the BR buffer was adjusted by addition of 0.20 mol/L sodium hydroxide solution. All other chemicals used for the experiments were of analytical grade (Merck, Darmstadt, Germany) and were used without further purification. Distilled water obtained from a MonoDest 3000E system (Brand, Wertheim, Germany) was used throughout the experiments.

### 2.2. Instrumentation

Instrumentation for chronopotentiometry consisted of automatic stripping analyser (M1 analyser) of domestic construction [46]. The analyser was coupled to an Epson LQ-570 printer (Suwa, Nagano, Japan). The electrochemical cell consisted of glass vessel with tapered bottom volume of 50 mL, three-electrode system, and electrical stick stirrer. Glassy carbon disc electrode (total surface area of 7.07 mm^2^) was used as a working electrode, but also as an inert support for TFME. A platinum wire (diameter = 0.7 mm, length = 7 mm) was used as a counter electrode, while the reference was Ag/AgCl (KCl, 3.50 mol/L) electrode. All values of the potential were shown versus Ag/AgCl (KCl, 3.5 mol/L) reference electrode. A digital pH meter model MA 5705 (Iskra, Kranj, Slovenia) with combined glass electrode was used for all pH measurements. An ultrasonic bath (Iskra, Kranj, Slovenia) with working frequency of 30 Hz and power of 500 W was used after the polishing procedure.

### 2.3. Preparation and Maintenance of the Working Electrodes

#### 2.3.1. Glassy Carbon Electrode

After each finished set of experiments the surface of the GCE was cleaned with filter paper wetted firstly with acetone and then with distilled water. For maintenance of the good quality of the glassy carbon surface, whenever the sensitivity dropped off, or after prolonged disuse of GCE, the polishing procedure was performed using aluminium oxide slurry, gained by mixing aluminium oxide (grain size 0.5 *μ*m, Merck, Darmstadt, Germany) with distilled water, on a special cotton panel, until a mirror-like surface was obtained. For removing residual particles, the electrode was first wiped with filter paper wetted with acetone and then with distilled water, and hereupon it was sonicated in a mixture of distilled water and ethanol (1 : 1, v/v) for 10 minutes. Afterwards, electrochemical pretreatment was performed by chronopotentiometric cycling between −0.70 V and 0.70 V (10 cycles) applying the current of 7.0 *μ*A, in 0.01 mol/L sulphuric acid. The unmodified GCE prepared this way was used as an inert support for TFME and as bare GCE for comparison purposes.

#### 2.3.2. Thin Film Mercury Electrode

Deposition of the thin mercury film was performed potentiostatically at the potential of −0.40 V for 4 min, from the solution containing 0.02 mol/L hydrochloric acid and 0.10 g/L of Hg^2+^ ion. The working electrode could be used for approximately 50 analyses, after which the film was removed, and deposition was repeated by the same procedure.

### 2.4. Samples and Sample Preparation

#### 2.4.1. Pesticide Formulations

Pesticide formulations of imidacloprid: Confidor 200 SL (Bayer CropScience, Monheim, Germany), Confidor 70 WG (Bayer CropScience, Monheim, Germany), Prestige 290 FS (Bayer CropScience, Monheim, Germany), Gat Go 20 OD (GAT Microencapsulation AG, Ebenfurth, Austria), Imidor 70 WS (Stockton Chemical, Florida, USA), and Kohinor 200 SL (Celsius Property, Amsterdam, Netherlands) were purchased from the local agricultural supplier (Novi Sad, Serbia). Due to high concentration of imidacloprid in the commercial formulations, appropriate dilution was required to obtain a suitable range of concentrations for chronopotentiometric analysis. The proper volume or weight of each sample was transferred to a 250 mL calibrated flask and filled with distilled water to accomplish around 0.5 g/L of imidacloprid. For chronopotentiometric analysis the appropriate volume of this solution was transferred to the electrochemical cell so that the final concentration of imidacloprid was in the range from 2 mg/L to 20 mg/L.

#### 2.4.2. River Water Samples

River water samples were collected in plastic bottles from the River Danube at five different locations on the territory of Novi Sad (Serbia) and were stored in the dark at 4°C until the analysis. Samples were taken in the urban recreational zone at the following sites: Petrovaradin fortress (1254 km of the flow), “Štrand” beach (1257 km of the flow), fishing weekend resort (1258 km of the flow), weekend resort “Kamenjar” (1263 km of the flow), and beach “Mačkov Prud” (1266 km of the flow).

A volume of 250 mL of each sample was filtered through membrane syringe filter with pore diameter of 0.45 *μ*m (Chromafil®Xtra PET-45/25, Macherey-Nagel, Düren, Germany). For performing chronopotentiometric analysis, 5 mL of water sample was added to the electrochemical cell filled with 15 mL of supporting electrolyte and analysed according to the previously optimized conditions. Filtered water samples were spiked with imidacloprid standard solution, and the final imidacloprid concentration in spiked water samples was 1 mg/L.

For performing LC-MS/MS analysis [47], extraction and preconcentration of previously filtered river water samples were performed by solid-phase extraction (SPE) method with Supelco, Supel*™*-Select HLB cartridges (200 mg, 6 mL), preconditioned with 5 mL of methanol and 5 mL of distilled water. Blank or spiked water samples (250 mL) were loaded on the cartridges at the rate of 3–5 mL/min by using water vacuum pump. After passing the sample, the cartridges were washed with 10 mL of distilled water and air dried for 10 minutes, and the analyte was eluted with 5 mL of methanol. The eluate was brought to dryness under a gentle nitrogen stream. The residue was dissolved in the 0.25 mL of initial mobile phase, and 10 *μ*L was injected into LC-MS/MS system. Quantification of imidacloprid was performed by means of the calibration curve method.

### 2.5. Chronopotentiometric Measurement

Electrochemical cell filled with 20 mL of the analysed solution was used for performing chronopotentiometric measurements. Dissolved oxygen was removed from the solution by adding previously optimized concentration of the saturated solution of sodium sulphite [48] and stirring the solution for 30 s and 15 s in each successive cycle. After the deaeration period, in order to enable diffusive mass transfer in the vicinity of the working electrode, the solution was left to rest for 10 s, and the analytical step was performed by recording of chronopotentiogram in the appropriate negative potential window. All experiments were performed using five replicates at the ambient temperature (23–25°C).

### 2.6. Optimization and Validation Procedures

In order to achieve the optimum conditions for chronopotentiometric determination of imidacloprid, the influence of the most important experimental parameters on imidacloprid analytical signal, including type and pH of the supporting electrolyte, initial potential, and reduction current were studied. The experimental parameter that provided the highest, well defined, reproducible, and sharp analytical signal of the analyte was accepted as optimal. The method was validated with respect to linearity, limit of detection (LOD), limit of quantification (LOQ), precision, selectivity, accuracy, and robustness according to US EPA guidelines [45]. Applicability and practical usage of the proposed method were verified by analysing real river water samples as well as pesticide formulations containing imidacloprid.

### 2.7. LC-MS/MS Analysis

In order to check the accuracy of the proposed chronopotentiometric method, LC-MS/MS method was also used to quantify imidacloprid content in river water samples extracted as described in Section 2.4.2. Chromatographic analysis was performed using an Agilent 1200 Series liquid chromatograph (Agilent Technologies Inc., Santa Clara, CA, USA) equipped with triple quad mass spectrometer Agilent 6410 (Agilent Technologies Inc., Santa Clara, CA, USA). Separation was achieved using XBridge C18 column (150 × 3 mm) with 3.5 *μ*m particle size (Waters, Milford, USA) maintained at 40°C. The mobile phase consisted of 0.1% (v/v) formic acid in methanol (A) and 0.1% (v/v) formic acid in water (B), with a flow rate of 0.5 mL/min. The gradient used started with 70% of mobile phase B during 2-minute hold constant, followed by a linear gradient reaching 50% B after 15 minutes, kept constant for 4 minutes, and finally decreased to 30% B after 20 minutes and kept on 30% for 6 minutes. Mass spectrometer was operated in multiple reactions monitoring mode for mass analysis of positive ions generated by electrospray ionization. The operating parameters for the mass spectrometer were as follows: heater gas temperature of 350°C and vaporization temperature of 250°C. Nitrogen was used as a nebulizer gas at 50 psi and flow rate of 5 L/min, capillary voltage of 3500 V, and charging voltage of 2000 V. For quantification of imidacloprid two precursor-to-product ion transitions were chosen 256.0-208.7 and 256.0-174.6. MassHunter Workstation software (Agilent Technologies, Santa Clara, CA, USA) was used for the control of equipment, data acquisition, and analysis.

## 3. Results and Discussion

### 3.1. Influence of the Type and pH of the Supporting Electrolyte

The selection of a suitable supporting electrolyte is considered as an essential step in electrochemical studies because its composition and pH can significantly affect ongoing electrochemical reactions [49]. The dependence of chronopotentiometric signals of imidacloprid on the type of the supporting electrolyte was evaluated using BR buffer, 0.10 mol/L citrate buffer, 0.10 mol/L phosphate buffer, 0.01 mol/L hydrochloric acid, 0.01 mol/L sulphuric acid, and 4.50 g/L sodium sulphite. In all studied electrolytes imidacloprid exhibited a single reduction wave on TFME from −0.97 to −1.04 V and on GCE from −1.18 to −1.28 V. In reversible potential scan, the absence of oxidation peak indicated that the electrode reactions are irreversible. Among the studied electrolytes, the highest sensitivity, well-defined reduction peak with good reproducibility was achieved using BR buffer, so this buffer was chosen as a supporting electrolyte. In order to choose optimal pH value of the BR buffer, chronopotentiograms of fixed concentrations of imidacloprid (1, 10, and 20 mg/L), with varying pH value of the buffer in the range from 2 to 10, were recorded, while other parameters of the analysis were kept constant. In general, higher sensitivity towards imidacloprid electroreduction was achieved on TFME in comparison to GCE.

**Figure 2:**
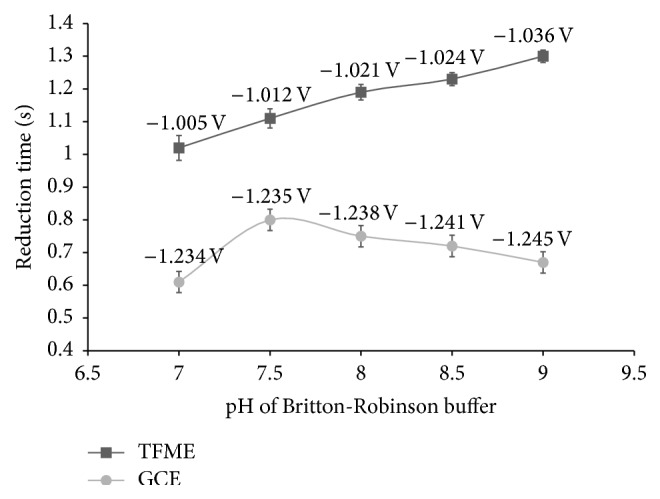
Effect of pH of BR buffer on the reduction time (s) and reduction peak potential (V) on TFME and GCE. Concentration of imidacloprid 10** **mg/L, mean ± 2SD, *n* = 5.

**Figure 3:**
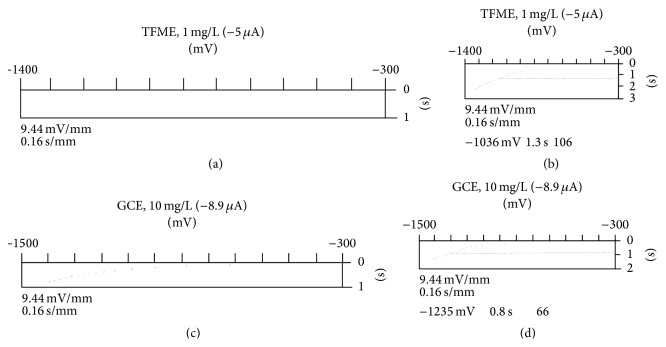
Chronopotentiograms of imidacloprid on a TFME ((a) *c* = 0 mg/L; (b) *c* = 1 mg/L, *i* = −5 *μ*A) and on GCE ((c) *c* = 0 mg/L; (d) *c* = 10 mg/L, *i* = −8.9 *μ*A).

In the pH range from 2 to 6 signal of imidacloprid was not observed on both working electrodes, since the ending potential was not reached due to a blockage of the electrode. In alkaline buffers (pH > 9) sufficient sensitivity was not achieved. Thus, a well-defined reduction wave of imidacloprid was obtained in narrow pH limit of the BR buffer from 7 to 9 (Figure 2). Within this pH range, imidacloprid signal on TFME slightly increased with pH increase, while on GCE reached maximum at pH 7.5 and then slightly decreased. In addition, on both working electrodes, the reduction peak potential shifted negatively with increasing of pH of BR buffer (Figure 2). As the most appropriate supporting electrolyte for TFME the BR buffer at pH 9.0 was chosen, while in the case of GCE pH 7.5 was more appropriate. Reproducibility of the imidacloprid analytical signals was very good (TFME, RSD = 1.39%, *n* = 5; GCE, RSD = 2.08%, *n* = 5). Chronopotentiograms recorded in BR buffers containing 10 mg/L of imidacloprid at pH 9 and pH 7.5 on TFME and GCE, respectively, are shown in Figure 3.

### 3.2. Influence of the Initial Potential

Influence of the initial potential on insecticide reduction time using TFME was investigated in the solution containing 1 mg/L of imidacloprid and by applying reduction current of −5.0 *μ*A. Well defined signals occurred in the range of initial potential from −0.12 V to −0.85 V. Values of the initial potential higher than −0.09 V led to permanent damage of mercury film, while at more negative values the analyte could not be detected. When GCE was used, investigated concentration of imidacloprid was 10 mg/L and reduction current of −8.9 *μ*A. Initial potentials more positive than −0.25 V provided outstretched chronopotentiograms. Well defined peaks of imidacloprid were detected in the range of initial potential from −0.25 to −1.00 V. According to the height and reproducibility of imidacloprid signal, values of initial potential chosen as suitable were on −0.18 V (RSD = 0.02%, *n* = 5) and −0.25 V (RSD = 2.55%, *n* = 5) for TFME and GCE, respectively. Applied values of the final potentials were for TFME −1.35 V and for GCE −1.42 V. By applying more negative values than accepted, outstretching of chronopotentiograms occurred, and analysis was significantly prolonged.

### 3.3. Influence of the Reduction Current

In chronopotentiometric analysis, the reduction current is one of the most important parameters of the analysis; it influences the height and sharpness of the analytical signal. Studied ranges of reduction current on TFME for solutions containing 1 and 5 mg/L were from −2.3 to −10.1 *μ*A and from −3.2 to −27.6 *μ*A, respectively. The reduction time of imidacloprid exponentially decreased with more negative values of reduction current for both investigated concentrations of imidacloprid (*c* = 1 mg/L: *τ*_red_ = 4.8384e^0.2918*I*^, *r* = 0.9985; *c* = 5 mg/L: *τ*_red_ = 2.2576e^0.1182*I*^, *r* = 0.9974). Similar correlations were observed on GCE for studied concentrations of 10 mg/L (*τ*_red_ = 6.3775e^0.2061*I*^, *r* = 0.9973) and 30 mg/L (*τ*_red_ = 6.4065e^0.1068*I*^, *r* = 0.9988), with ranges of reduction current from −6.6 to −10.4 *μ*A and from −8.8 to −31.8 *μ*A, respectively. Based on criterion of rectilinear sequence of the dependence *I* · *τ*_red_^1/2^ = *f*(*I*), the appropriate intervals of reduction current that should be applied for investigated concentrations on TFME (1 and 5 mg/L) are from −3.8 to −9.4 *μ*A and from −10.1 to −27.6 *μ*A, respectively. On GCE, optimal intervals of reduction current for concentrations of 10 and 30 mg/L are from −6.6 to −9.7 *μ*A and from −14.0 to −26 *μ*A. Generally, for detecting lower concentrations lower absolute values of the current are required and* vice versa*. Thus, the appropriate value of reduction current that should be applied in the analysis should be selected from the above mentioned ranges, depending on the working electrode and studied concentration. Given the wide range of the current at which the signal of imidacloprid was obtained, the reduction potential did not vary significantly. Imidacloprid reduction wave on TFME was appeared at a potential range from −0.97 V to −1.12 V (RSD = 4.20%, *n* = 45), while somewhat lower deviation of reduction potential was observed on GCE from −1.20 to −1.28 V (RSD = 1.59%, *n* = 45).

### 3.4. Validation of Method

#### 3.4.1. Linearity

Linearity was estimated by analysing standard solutions containing imidacloprid in the range from 0.8 to 30.0 mg/L on TFME and on GCE from 7.0 to 70.0 mg/L. In chronopotentiometry characteristic dependence of the transition time versus concentrations implies polynomial characteristic, but analytical methodologies tend to perform quantification in linear range. Accordingly, three individual concentrations ranges were considered in order to cover broader range of imidacloprid contents. For every studied concentration range different reduction current was applied and experiments were performed in five replicates. Calibration plots, standard deviations of the intercept (S_*a*_), and slope (S_*b*_) obtained by the least squares linear regression method, with applied reduction current are summarized in Table 1. Under the optimal experimental conditions, very good linear correlations were obtained for three concentration ranges on both working electrodes, with correlation coefficients in the range from 0.9975 to 0.9989. As reduction current significantly influences the method sensitivity, different applied reduction current was resulting in different slopes of defined calibration curves.

#### 3.4.2. Limit of Detection and Limit of Quantification

The limit of detection (LOD) and limit of quantification (LOQ) values were calculated by the following formulas: LOD = 3.3S_*a*_/*b* and LOQ = 10S_*a*_/*b* [50], where *S*_*a*_ is the standard deviation of the intercept, and *b* is the slope of the calibration curve, both defined for the LOD concentration range (0.8–2.0 mg/L by using TFME, and 7.0–15.0 mg/L by using GCE). The calculated values of LOD were 0.17 mg/L for TFME and 0.92 mg/L for GCE and of LOQ were 0.51 mg/L for TFME and 2.80 mg/L for GCE. In comparison to pulse polarographic and voltammetric techniques, chronopotentiometric method proposed in this work showed lower sensitivity towards imidacloprid determination (Table 2). However, it should be considered that application of previously mentioned techniques is limited to use of HMDE. Numerous disadvantages that are related to these electrodes can be overcome by using mercury film electrodes. They can be of fairly small size, provide larger surface-to-volume ratio, are mechanically more stable than mercury drops, and require only minute quantities of mercury and the possibility for in situ analysis [51]. Multitude of modified GCEs also contributed to increase of sensitivity towards imidacloprid determination, but these electrodes require long and complicated preparation procedures, and some of them (*β*-CDP/rGO/GCE and PCz/CRGO/GCE) did not find its practical application [32, 42]. Bismuth, silver-amalgam based electrodes, CPEs, and GCEs represent also attempts to avoid the use of mercury in imidacloprid determination, but it is evident that these electrodes did not show adequate sensitivity (Table 2). From the aspect of this study, since GCE did not show respectable sensitivity, chronopotentiometry with use of TFME represents a quick and easy access in imidacloprid determination, and, with prior concentration of the sample by SPE, it shows to be more sensitive in comparison with other reported electrochemical methods (Table 2). In fact, by using the SPE method in analysis of real samples, as it was described for LC-MS/MS analysis, detection limit for thin film mercury electrode could be improved up to 0.01 mg/L.

#### 3.4.3. Precision

Precision was estimated by values of repeatability (intraday precision) and reproducibility (interday precision) for two concentrations of imidacloprid. Repeatability was determined by the value of relative standard deviation (RSD) for five replicate analyses of identically prepared standard solutions of imidacloprid within the same day. Reproducibility was determined by calculating the RSD value obtained by determining imidacloprid on five successive days. Investigated concentrations on TFME were 1 and 5 mg/L, applying reduction currents of −5.0 and −10.1 *μ*A, respectively. On GCE concentrations of 10 and 30 mg/L were investigated, while applied reduction currents were −8.9 and −16.7 *μ*A, respectively. In all experiments related to estimation of precision gained values of RSD were less than 3.73%, indicating good precision of the presented method independently of the working electrode applied.

#### 3.4.4. Selectivity

The selectivity of the presented method was tested by addition of various inorganic ions and two reducible herbicides metribuzin and metamitron to the solution containing fixed concentration of imidacloprid. Selected inorganic ions (K^+^, Na^+^, Ca^2+^, Mg^2+^, SO_4_^2−^, Cl^−^, HCO_3_^−^, NO_3_^−^, and Fe^2+^) which could be present in environmental water samples were added in the concentration ratios (imidacloprid : interferent) of 1 : 0.1, 1 : 1, 1 : 10, and 1 : 50. The influence of inorganic ions on analytical signal of imidacloprid was tested on both working electrodes, while the influence of herbicides was investigated using TFME. Considering the precision of the method, a compound was considered to interfere seriously if it provoked the change of imidacloprid signal more than 5%. Tested concentrations of imidacloprid on TFME and GCE were 1 mg/L and 10 mg/L, respectively. The majority of tested ions (K^+^, NO_3_^−^, Cl^−^, HCO_3_^−^, and SO_4_^2−^) in 50-fold excess provoked minor decrease of analytical signal, while Ca^2+^, Mg^2+^, and Na^+^ slightly increased imidacloprid signal, with maximum change of 4.96%. Concerning Fe^2+^ ions, its presence at concentrations less than 10 mg/L led to signal change within the 5% limit, while higher concentrations slightly decreased the analytical signal. This phenomenon could be attributed to a Fe^2+^ reduction peak [52], which interfered with imidacloprid determination. This fact could somewhat reduce the selectivity of the method, but when considering the possibility of using developed method for determination of imidacloprid in drinking water, where Fe^2+^ ion is present in lower concentrations (0.7 mg/L) [53], the interference of this ion could be excluded. Caution should be made in the direct implementation of the developed method to groundwater samples, where increased concentration of mentioned ion is expected. In that case, elimination of Fe^2+^ ions is necessary using cationic cartridges. As concerned for the presence of herbicides, 10-fold excess concentrations of metribuzin and metamitron and 50-fold excess concentration of metamitron provoked minor increase of imidacloprid analytical signal (less than 5%). In the case of 50-fold excess of metribuzin concentration, the signal change was slightly more (6.2%).

#### 3.4.5. Accuracy

Estimation of proposed chronopotentiometric method accuracy was based on means of analyses of imidacloprid standard solutions. Supporting electrolyte spiked with known amount of imidacloprid (1 and 5 mg/dm^3^) was analysed under optimized chronopotentiometric procedure.

Recovery test was performed in three replicates for both examined concentrations of insecticide. The percentage analytical recovery values were calculated by comparing concentrations determined from the spiked supporting electrolyte with actual added concentrations of imidacloprid. Good values of the mean recoveries of 97.3% and 98.1%, for 1 mg/dm^3^ and 5 mg/dm^3^ of imidacloprid, respectively, confirmed the accuracy of the proposed method and offered the promising evidence that the developed method could be used in the analysis of real samples.

For additional check of the accuracy of the developed method, LC-MS/MS parallel analyses of river water samples were done. Obtained results are given in Section 3.5.2.

#### 3.4.6. Robustness

The robustness of the developed method was evaluated by investigating the effect of small variations in pH value of supporting electrolyte (±0.2), the initial potential (±0.05 V), and reduction current (−8.2 ± 0.3 *μ*A) on the recovery of analyte. Recoveries for imidacloprid (5 mg/L) under all variable conditions were in the range of 97.1–99.4%. Constancy of the transition time with deliberated small changes in the experimental parameters indicated a very good robustness of the proposed method.

### 3.5. Analytical Applications

#### 3.5.1. Application to Commercial Formulations

In order to investigate the validity of the presented chronopotentiometric method, commercial pesticide formulations containing imidacloprid as active component were analysed. Considering the fact that no extraction steps were performed prior to analysis, except the appropriate dilution steps, no interferences were observed during the analysis. When GCE was used in the analysis of liquid formulations, due to presence of various additives that improve the formulation properties, fouling of the glassy carbon surface occurred, and gained signal of the analyte was not reproducible. Thus, between two analyses it was necessary to perform electrochemical cleaning of the glassy carbon surface. The procedure consisted of ten in situ consecutive cycles of potential alternation from −0.25 V to −1.42 V, by the current that was applied regularly for the analysis, which enables that every analysis could be performed on freshly activated surface [54]. All experiments were performed using three replicates, and imidacloprid was quantified by the standard addition method. Obtained results are presented in Table 3. Good correlation between amounts determined and declared or added, as well as low values of RSD reflect the high accuracy and precision, indicating that presented chronopotentiometric method can be used as a routine tool for control of imidacloprid content in commercial pesticide formulations, without interferences from inactive ingredients.

#### 3.5.2. Application to River Water Samples

Due to its high sensitivity, the present method using TFME was applied for quantification of imidacloprid content in five river water samples. Experiments were performed using three replicates, and imidacloprid content was determined by the standard addition method. By direct analysis, river water samples did not show any measurable quantities of the analyte, so they were spiked with the same concentration of the imidacloprid (1 mg/L). Moreover, the chronopotentiograms profile excluded the presence of interfering electroactive compounds in the analysed solution. Obtained results with RSD and recovery values are presented in Table 4. A parallel analysis of river water samples by LC-MS/MS method was done for additional accuracy check. According to the results from Table 4, obtained recovery values for presented chronopotentiometric method were between 93.64 and 97.95%, which are acceptable for studied concentration [55]. High reproducibility of the proposed method was indicated by the maximum RSD value of 2.56%. Moreover, the results of the proposed method were comparable to those obtained from the LC-MS/MS method with no significant difference between the two methods (paired *t*-test at the 95% confidence level gave |*t*_calculated_| = 1.28 < *t*_critical_ = 2.78, with 4 degrees of freedom). Based on obtained results, it is obvious that the presented method provides a good alternative for imidacloprid quantification in environmental samples with adequate sample preparation.

## 4. Conclusions

Chronopotentiometry in combination with TFME and GCE was used for the first time to develop a simple, rapid, and cheap electrochemical method for imidacloprid determination. During imidacloprid reduction, one well defined reductive peak of analyte appeared at both electrodes. The effects of the supporting electrolyte, pH, initial and final potential, and reduction current on the analytical signal of imidacloprid was investigated. In addition, parameters related to linearity and precision were also estimated. Obtained LOD values are comparable to values reported in the literature for other electrochemical methods. Linearity and precision of the method were adequate, and correlation coefficients were higher than 0.9975, while precision gained values of RSD were up to 3.73%. High recoveries of spiked samples confirmed the accuracy of the method. The method proved as selective, since the majority of possible interferents did not influence the analytical signal of the analyte. The validity of the method was confirmed by direct analysis of commercial formulation containing imidacloprid with no significant differences between declared values by manufacturer and values found by the presented chronopotentiometric method. Due to higher sensitivity, the method using TFME was also applied on spiked river water samples, and the results were in good agreements with those obtained by reference LC-MS/MS method. In addition, TFME represents a suitable alternative to a HMDE, due to its advantages, which include stability of thin film, minimal consumption of mercury, easy preparation and maintenance procedure, and suitability for on-site analysis. Thus, simplicity, rapidity, and low cost are the main characteristics that distinguished the presented method from previously reported electrochemical methods for imidacloprid determination. Moreover, simple preparation of working electrode and short analysis time of real samples are clear advantages of the presented method. Hence, this method can undoubtedly serve as an alternative to complicated chromatographic techniques for routine imidacloprid determination in environmental water samples.

## Figures and Tables

**Table 1:** Linear ranges for chronopotentiometric determination of imidacloprid using thin film mercury and glassy carbon electrode.

Electrode	Concentration range [mg/L]	Reduction current [*μ*A]	Slope	Intercept	S_*b*_^a^	S_*a*_^b^	*r*
Thin film Mercury Electrode	0.8–2.0	−3.8	0.257	0.898	0.0187	0.0131	0.9989
	2.0–10.0	−6.2	0.132	0.390	0.0020	0.0195	0.9975
	10.0–30.0	−16.8	0.086	−0.280	0.0022	0.0123	0.9980

Glassy Carbon Electrode	7.0–15.0	−6.6	0.046	0.329	0.0031	0.0129	0.9976
	10.0–40.0	−14.0	0.032	0.038	0.0007	0.0012	0.9987
	40.0–70.0	−18.4	0.051	−0.730	0.0002	0.0220	0.9986

^a^S_*b*_ represents the standard deviation of the slope in s·L/mg, *n* = 5.

^b^S_*a*_ represents the standard deviation of the intercept in s, *n* = 5.

**Table 2:** Comparison of limit of detection (LOD) of the presented chronopotentiometric method with previously reported electrochemical methods for imidacloprid determination.

LOD [mg/L]	Technique	Electrode	Reference
0.0030	Differential pulse polarography	HMDE	[33]
0.0041	Square wave adsorptive stripping voltammetry	HMDE	[34]
0.0051	Differential pulse voltammetry	*β*-CDP/rGO/GCE	[42]
0.0128	Linear sweep voltammetry	Prussian blue/MWNT/GCE	[40]
**0.0134**	**Chronopotentiometry with SPE**	**TFME**	**This study**
0.0256	Cyclic voltammetry	*β*-CDP/rGO/GCE	[42]
0.0563	Cyclic voltammetry	PCz/CRGO/GCE	[32]
0.0640	Differential pulse voltammetry	nAgn_f_/nTiO_2_n_f_/GCE	[41]
0.0716	Differential pulse voltammetry	CuPc/CCE	[44]
0.1023	Linear sweep voltammetry	Imprinted PoPD-RGO/GCE	[43]
0.1125	Differential pulse voltammetry	PCz/CRGO/GCE	[32]
0.1611	Cyclic voltammetry	nAgn_f_/nTiO_2_n_f_/GCE	[41]
**0.1682**	**Chronopotentiometry**	**TFME**	**This study**
0.2378	Amperometry	nAgn_f_/nTiO_2_n_f_/GCE	[41]
0.2700	Square wave voltammetry	Hg(Ag)FE	[39]
0.5200	Differential pulse voltammetry	CPE	[37]
0.7300	Differential pulse voltammetry	BiFE	[38]
**0.9254**	**Chronopotentiometry**	**GCE**	**This study**
7.7000	Cyclic voltammetry	GCE	[35]

HMDE: hanging mercury drop electrode; *β*-CDP/rGO/GCE: *β*-cyclodextrin polymer/functionalized reduced-graphene oxide/modified GCE; TFME: thin film mercury electrode; Prussian blue/MWNT/GCE: Prussian blue multiwalled carbon nanotubes modified GCE; PCz/CRGO/GCE: poly(carbazole)/chemically reduced graphene oxide modified GCE; Hg(Ag)FE: silver-amalgam film electrode; nAgn_f_/nTiO_2_n_f_/GCE: nanosilver Nafion/nano-TiO_2_ Nafion modified glassy carbon electrode; CuPc/CCE: copper(II) phthalocyanine modified carbon ceramic electrode; imprinted PoPD-RGO/GCE: imprinted poly(o-phenylenediamine) membranes/reduced graphene oxide modified GCE; CPE: carbon paste electrode; BiFE: bismuth film electrode; GCE: glassy carbon electrode.

**Table 3:** Results obtained for the analysis of commercial formulations by the proposed chronopotentiometric method.

Commercial pesticide formulation	Imidacloprid content claimed by the manufacturer	Determined by the proposed chronopotentiometric method	
		TFME	GCE
	Imidacloprid content [g/L]		
Prestige 290 FS	140.00	144.17 ± 1.60 (102.98)^a^	134.27 ± 2.52 (95.91)
Confidor 200 SL	200.00	199.71 ± 1.59 (99.86)	197.02 ± 1.39 (98.51)
Kohinor 200 SL	200.00	202.02 ± 0.82 (101.01)	203.33 ± 1.35 (101.67)
Gat Go 20 OD	200.00	204.22 ± 1.98 (102.11)	196.46 ± 0.72 (98.23)

	Imidacloprid content [g/kg]		
Confidor 70 WG	700.00	697.98 ± 2.07 (99.71)	685.72 ± 1.19 (97.96)
Imidor 70 WS	700.00	708.42 ± 1.29 (101.20)	712.19 ± 1.10 (101.74)

^a^Mean value ± RSD (recovery, %), *n* = 3.

**Table 4:** Results obtained for the analysis of spiked river water samples by the proposed chronopotentiometric method and reference LC-MS/MS method.

Sample	Added [mg/L]	Proposed chronopotentiometric method		LC-MS/MS method	
		Found [mg/L]	Recovery [%]	Found [mg/L]	Recovery [%]
1	1.00	0.98 ± 1.64^a^	97.94	1.03 ± 0.32^a^	103.20
2	1.00	0.98 ± 2.56	97.95	0.96 ± 6.78	95.92
3	1.00	0.94 ± 0.79	93.64	0.99 ± 2.00	97.87
4	1.00	0.96 ± 2.04	96.26	0.94 ± 1.96	93.39
5	1.00	0.95 ± 0.64	95.19	1.02 ± 4.50	101.77

^a^Mean value ± RSD, *n* = 3.
